# Proadi-SUS: analysis of financial resources in the three-year periods 2009–2011, 2012–2014 and 2015–2017

**DOI:** 10.11606/s1518-8787.2023057004385

**Published:** 2023-09-14

**Authors:** Julia Amorim Santos, Luciana Palhares, Aquilas Mendes

**Affiliations:** I Universidade de São Paulo Faculdade de Saúde Pública Programa de Pós-Graduação em Saúde Pública São Paulo SP Brasil Universidade de São Paulo. Faculdade de Saúde Pública. Programa de Pós-Graduação em Saúde Pública. São Paulo, SP, Brasil; II Universidade Estadual de Campinas Faculdade de Educação Programa de Pós-Graduação em Sociologia da Educação Campinas SP Brasil Universidade Estadual de Campinas. Faculdade de Educação. Programa de Pós-Graduação em Sociologia da Educação. Campinas, SP, Brasil; III Pontifícia Universidade Católica de São Paulo Programa de Estudos Pós-graduados em Economia Política São Paulo SP Brasil Pontifícia Universidade Católica de São Paulo. Programa de Estudos Pós-graduados em Economia Política. São Paulo, SP, Brasil

**Keywords:** Health Policy, Public-Private Partnerships, Tax Exemption

## Abstract

**OBJECTIVE:**

To characterize the tax exemption resources used in the Support Program for Institutional Development of the Unified Health System (Proadi-SUS) in the 3-year periods 2009–2011, 2012–2014, 2015–2017, considering the total volume of resources linked to the debate on tax expenditures on health and the constitution of a “new form of philanthropy” in the sector.

**METHODS:**

To understand the philanthropic sector, tax expenditures between 2001 and 2017 were analyzed. To evaluate the resources used in the program, the values of projects and areas of activity were examined.

**RESULTS:**

A real increase in the values of general tax expenses and tax expenses referring to the philanthropic sector was found. There was also a real increase in the program’s resources. A total of 407 projects were carried out, amounting to R$ 3.4 billion for the period. An analysis of the average value of the projects shows an increase in values for all hospitals included in the program, with the exception of one of the institutions. In the 2009–2011 and 2012–2014 periods, the area with the highest number of projects and the most resources was “Management techniques and operation in health services”. In the 3-year period 2015-2017, however, the sector that received the most investments and the largest number of projects developed was “Human Resources Training”.

**CONCLUSION:**

The program characterizes a different expression of the public-private partnership in the health sector linked to the principles of the new public management. As a development for future investigations, a qualitative characterization of the projects developed and the actions’ impact on the public sector demands is necessary.

## INTRODUCTION

According to the Ministry of Health (MH), the Support Program for Institutional Development of the Unified Health System (Proadi-SUS) is a policy aimed at strengthening the Unified Health System (SUS) to be conducted in partnership with philanthropic hospitals of recognized quality^1,2^. This program is financed from tax expenditures, which are considered indirect expenditures from the Federal Government’s tax exemption. The tax expenditure policy aims to meet certain economic and social objectives and constitutes an exception to the Reference Tax System. The government assigns tax relief to stimulate certain areas, considered essential, reducing potential collection, but, consequently, increasing the taxpayer’s economic availability^3^.

The principle of constituting the institutional arrangement for implementing Proadi-SUS was established in the 1990s, during the President Fernando Henrique Cardoso’s administration. Through Decree No. 2,536 of 1998, the possibility for philanthropic entities to access tax exemptions by carrying out projects aimed at the SUS was established for the first time in the health sector. Such initiatives go beyond the provision of assistance services as established by traditional philanthropy regulated by Law No. 8,742, of 1993, which provides for the provision of 60% of resources in assistance actions for the SUS. From that moment on, the possibility of developing projects in other areas of action was instituted, limited to 30% of the amount used with assistance actions^2^. A document published by the institutions that make up the program refers to the emergence of a new level in the relationship between hospitals and the state sphere, with the institution of a new form of philanthropy^4^.

This form of philanthropy, in addition to resizing the percentage of assistance actions as mentioned above, also predicts a difference in cost parameters. In traditional philanthropy, the SUS table is based on the price, while the Proadi-SUS allows hospitals to carry out actions based on the market table.

In addition to contributions with resources for assistance activities and the expansion of actions with the implementation of projects, it can be said that this experience is characterized as a “new form of philanthropy” because it is in line with what was recommended by the 1995 State Reform, which proposed changes in state administration and new management proposals^5^, in addition to introducing new criteria for granting philanthropy certificates to hospitals that were considered strategic. An institutional arrangement has been instituted with a new form of public-private relationship with the regulation of the business community to develop social work in the health sector^6,7^.

The literature also indicates that in the years 2000/2010, including through guidelines from international organizations such as the World Bank, there was an expansion of the managerial logic and the relationship between the various actors to finance, monitor, provide and use health services. The concept of “social entrepreneurship” was linked to public policy strategies and part of the responsibility for implementing State policies was attributed to sectors other than the State, such as the business community^8,9^. Therefore, the program’s guidelines are in line with the rationale of the *new public management*^10,11^. One of the central focuses was on adapting and transferring managerial knowledge developed in the private sector to the state sector.

Although the guarantee of tax exemptions through the execution of projects was instituted in the late 1990s, the regulation for the start of project activities to support the SUS by philanthropic entities was only consolidated in 2006, in dialogue with the advance of the management logic in the sector after the 2000s. The advance of this policy for the health sector was also marked by the new regulation of the Certificate of Charitable Social Assistance Entities (Cebas), in 2006, through Decree No. 5,895. This decree regulated a practice that had been carried out for eight years, in which some hospitals considered strategic already enjoyed tax exemption through this other philanthropic arrangement^2^. Thus, it should be noted that one of the justifications for the creation of Proadi-SUS was due to the need for greater regulation by the state sector in relation to this new form of philanthropy.

The program was instituted by Law No. 12,101/2009, which provides for Cebas and regulates the procedures for exemption from social contributions. It was based on this process that the design of projects to support the institutional development of the SUS was regulated in the following areas of action: I —technology incorporation evaluation studies; II — human resources training; III — research of public interest in health; and IV — management techniques and operation in health services^1,2,12^.

Thus, the Proadi-SUS was configured as a pioneering experience in this format, and programs like this are not found in the state management of the health sector previously. However, from 2012 onwards, programs were created in a similar format, such as the National Support Program for Oncological Care (Pronon) and the National Support Program for Health Care for Persons with Disabilities (Pronas/PCD)^13^, which also grant certification of philanthropy and tax exemption through the execution of projects in other areas, in addition to assistance. This fact can be considered an outstanding justification for the development of academic investigations, in addition to the fact that there is a shortage of assessments of the program^14,15^.

In addition to the justifications directly related to the program, there is also an indication in the literature of the importance of analyses on tax waivers and tax expenditures on health, with a certain scarcity of publications on this topic being observed again^16^. This topic is even more urgent because it is linked to the issue of SUS funding and the fight against underfunding in the public sector, stepped up as of 2016, with Constitutional Amendment No. 95^19,20^.

Salvador^17^mentions that in contemporary times the public fund enables the reproduction of capital, and the study of the budget should be considered an important element to understand social policy. For the author, in addition to a technical aspect, public funding reflects the correlation of social forces and the interests involved in the appropriation of state resources. In this way, analyzing Proadi-SUS funding must also permeate a broader investigation of social policies.

This article aims to characterize the tax exemption resources enjoyed in Proadi-SUS in the 3-year periods 2009–2011, 2012–2014 and 2015–2017. The analysis of these data represents an excerpt from the doctoral research that aimed to examine the program in question. In the doctoral thesis, a study is presented that encompasses the program’s articulation with social policies more broadly^21^, however, such analysis is not presented within the scope of this article.

## METHODS

Information was requested on Proadi-SUS for MH regarding the projects of its first 3-year periods (2009–2011, 2012–2014 and 2015–2017). This request was addressed both to the Department of Health Economics, Investments and Development (Desid) — responsible for managing the program — and through the Transparency Portal of the Comptroller General of the Union (CGU). Data on the projects were made available in spreadsheets sent to the researcher and through updates on the MH website^1^. As structured materials were shared in different ways, extensive work was required to organize the information to carry out the analysis.

To systematize the projects, the names, their description, the areas of activity and the values approved or executed were considered, depending on what information was made available. The following survey was established for analysis, according to the data made available through the spreadsheets that presented the financial resources: *values approved* for each project presented in the periods 2009–2011 and 2012–2014; *executed amounts* related to assistance projects for the 2012–2014 period; and *executed amounts* related to projects for 2015–2017.

After processing the information, inconsistencies and divergences were noticed in the available data. In the spreadsheet for the 2015–2017 period, there was no specification of the hospitals that carried out each project, only the value and name of the project were provided. Thus, it was still necessary to cross-check the information with the projects description published on the Ministry of Health website. As some projects were also not identified on this website, it was necessary to seek the reference of the hospital through the Management Committee minutes available on the institutional website of the MH^1^or in the publication Proadi-SUS^4^, carried out by the hospitals participating in the program. Projects disclosed on the MH website, but which were indicated in the financial expenses spreadsheet as not being executed, were disregarded. As there was an inaccuracy of nomenclature in these materials, those with similar titles and objectives were considered as the same project.

The researchers also found divergences in the references of the areas of activity, as compared to the information on the Ministry of Health website and in the project values spreadsheet. As indicated, the financial resources spreadsheet was used as a basis for the analysis, but in order to identify the divergences regarding the area of activity, the project description was read, making adjustments based on this analysis.

Tax expenditures and expenditures linked to the philanthropic sector in the period from 2001 to 2017 were also systematized. This information was accessed from the Statement of Tax Expenditures made available by the Ministry of Finance on the Federal Revenue website^22^. All data referring to current values were updated in real values, using the deflator of the Extended National Consumer Price Index (IPCA) of December 2017. This reference period was considered because it was the last year included in the analysis period of this work.

## RESULTS

For a more detailed analysis of the growth of the philanthropic sector in recent years, data on tax expenditures for the period 2001 and 2017 are presented in Table 1.

**Table 1:** Federal Government general tax expenditures and expenditures linked to the philanthropic sector in the period between 2001 and 2017, considering the annual percentage change.

Year	Total tax expense		Total spending linked to the philanthropic sector	
	
	Value in thousand *reais*	Annual percentage change (%)	Value in thousand *reais*	Annual percentage change (%)
2001	845,723		8,854	
2002	9,650,942	91.2	15,488	42.8
2003	10,864,218	11.2	96,335	83.9
2004	11,813,505	8	896,402	89.3
2005	16,135,205	26.8	1,693,394	47.1
2006	22,605,381	28.6	2,329,187	27.3
2007	29,302,590	22.9	3,409,711	31.7
2008	44,751,559	34.5	3,857,260	11.6
2009	62,578,136	28.5	4,789,882	19.5
2010	74,023,452	15.5	6,346,147	24.5
2011	80,365,722	7.9	5,887,764	-7.8
2012	106,962,736	24.9	6,928,435	15
2013	131,939,897	18.9	8,330,175	16.8
2014	206,245,036	36	17,885,947	53.4
2015	258,120,379	20.1	21,826,574	18.1
2016	263,247,117	1.9	23,478,916	7
2017	284,846,251	7.6	26,194,656	10.4

Source: Authors’ preparation based on Federal Revenue data (2017).

Data in *reais*, deflated from the December 2017 IPCA.

There is constant growth in values from year to year, with a higher increase in general tax expenditures by the Federal Government from 2003 to 2007. When analyzing tax expenditures related to the philanthropic sector, there was also significant and constant growth, with the exception of the passage from 2010 to 2011, which saw a 7.8% drop in value, but, on the other hand, there was an increase of 15% in the following year. Still referring to Table 1, examining philanthropy-related tax expenditures, a significant increase can be seen in the early 2000s. The amounts referring to philanthropy-related tax expenditures also had a significant increase between 2012 and 2015, with emphasis on 2014, which had a percentage change of 53.4%. When considering budgetary functions in relation to tax expenditures, there was a real increase in resources for social assistance, science and technology, culture, sports and leisure, education and health.

The six hospitals that made up the Proadi-SUS in the analyzed period are: Hospital Alemão Oswaldo Cruz (HAOC – SP); Hospital do Coração (HCor – SP); Hospital Israelita Albert Einstein (HIAE – SP); Hospital Moinhos de Vento (HMV – RS); Hospital Samaritan (SP); Hospital Sírio Libanês (HSL – SP). Based on the analysis of data from the projects developed and the tax exemption values linked to Proadi-SUS, it was found that, in the three 3-year periods, 407 projects were carried out, totaling a value of R$ 3.4 billion (Table 2).

**Table 2:** Indication of the number of projects and the tax exemption values of each of the institutions that make up the Proadi-SUS, considering the 3-year periods 2009–2011, 2012–2014 and 2015–2017, and the total reference for the period studied.

Institution Name	2009–2011		2012–2014		2015–2017	
		
	Number of projects	Value in thousand *reais*	Number of projects	Value in thousand *reais*	Number of projects	Amount in R$
Hospital Alemão Oswaldo Cruz	15	66,556	17	107,834	18	164,029
Hospital do Coração	27	50,103	33	88,671	28	126,683
Hospital Israelita Albert Einstein	32	274,780	41	442,417	23	727,123
Hospital Moinhos de Vento	4	50,530	7	105,928	18	188,689
Hospital Samaritano	21	33,473	26	88,711	14	123,569
Hospital Sírio Libanês	25	124,814	25	181,371	33	432,156
**Total per 3-year period**	**124**	**600,258**	**149**	**1,014,934**	**134**	**1,762,251**
**Program total**	**407 projects**					
	**3,377,444**					

Source: Authors’ preparation based on data from the Ministry of Health.

Data in *reais*, deflated from the December 2017 IPCA.

As can be seen in Table 2, in the first three years, from 2009 to 2011, 124 projects were developed, considering tax exemption resources in the amount of R$600.3 million. For the second 3-year period, from 2012 to 2014, 149 projects were created, considering tax exemption resources in the amount of R$ 1 billion. For the third period, from 2015 to 2017, 134 projects were prepared, considering tax exemption resources in the amount of R$ 1.8 billion. There is a real increase in program resources, with an annual change of 40.9% from the first to the second period and an annual change of 42.4% from the second to the third period.

When analyzing the number of projects and resources used by each of the hospitals in each 3-year period, a significant difference in the resources used and the number of projects developed by each of the institutions is identified.

Considering the total amount used by each of the hospitals, it is observed that 42.8% of the resources corresponded to the projects of the Hospital Israelita Albert Einstein and 21.9% of the resources referred to the projects of the Hospital Sírio Libanês. It is emphasized that the tax exemption value is calculated from the revenue of each hospital per year. This proportion is not corresponding as compared to the analysis of the number of projects carried out. Adopting the two hospitals referred to as a basis, it can be seen that 23.6% corresponded to the projects of the Hospital Israelita Albert Einstein and 20.4% to the projects of the Hospital Sírio Libanês. Thus, a differentiation between the average costs of projects is made explicit.

By discriminating the program’s tax exemption values per 3-year period, it is observed that all hospitals had a real increase in resources, with emphasis on the expansion of hospitals Israelita Albert Einstein and Sírio Libanês. Analyzing the average value of projects for each hospital, an increase in project values for all hospitals is observed when comparing the first, second and third 3-year periods, with the exception of Hospital Moinhos de Vento, which had a decrease in the average value from the second to the third period (Table 3).

**Table 3:** Average project value of each of the institutions that make up Proadi-SUS, considering the 3-year periods 2009–2011, 2012–2014 and 2015–2017, and the total reference for the period studied.

Institution Name	2009–2011	2012–2014	2015–2017	Total
			
	Value in thousands *reais*	Value in thousands *reais*	Value in thousands *reais*	Value in thousands *reais*
Hospital Alemão Oswaldo Cruz	4,437	6,343	9,112	6,768
Hospital do Coração	1,855	2,687	4,524	3,016
Hospital Israelita Albert Einstein	8,586	10,790	31,614	15,045
Hospital Moinhos de Vento	12,632	15,132	10,482	11,901
Hospital Samaritano	1,593	3,411	8,826	4,028
Hospital Sírio Libanês	4,992	7,254	13,095	8,895

Source: Authors’ preparation based on data from the Ministry of Health.

Data in *reais*, deflated from the December 2017 IPCA.

It is clear that the average value of projects at Hospital Israelita Albert Einstein is higher than that of other hospitals. Regarding the resources for the first three years, the tax exemption for this hospital corresponded to 45.5% of the resources available in the period, accounting for 25.8% of the projects carried out. As for the funds for the second 3-year period, the tax exemption for this hospital corresponded to 43.6% of the funds available in the period, accounting for 27.5% of the projects carried out. Regarding the resources for the third 3-year period, the tax exemption for this hospital corresponded to 41.3% of the resources available in the period, accounting for 17.2% of the projects carried out. Therefore, the average values of projects in the third period were significantly higher than in the other periods, with a variation of 72.8% from the average value of projects from the first to the third 3-year period.

It is also worth mentioning a significant difference in the percentage of resources and the number of projects carried out by Hospital Moinhos de Vento, with a significant increase in the number of projects in this hospital when the third period is compared to the first.

Proximity is observed between the percentage of projects and the percentage of resources at Hospital Alemão Oswaldo Cruz and Hospital Sírio Libanês. The hospitals that had the lowest average project value were Hospital do Coração and Hospital Samaritano.

The possibility was also opened, as of the second 3-year period, that the hospitals that were part of the program, and perhaps candidates for financing by the National Bank for Economic and Social Development (BNDES), could apply 5% of their financing by developing projects linked to Proadi-SUS. Therefore, in addition to the Proadi-SUS projects presented, a linkage of new projects with the BNDES was established. In the resource spreadsheet for the 2012–2014 period referring to projects linked to the BNDES, the execution of 11 projects was identified, in an amount corresponding to R$ 19.4 million (executed values). Of the hospitals that make up the program, only Hospital Israelita Albert Einstein adhered to this form in this 3-year period. It was possible to identify, through the Management Committee minutes, the existence of six projects developed by Hospital Israelita Albert Einstein and Hospital Sírio Libanês in this form, with approved amounts of R$ 28 million for the 3-year period 2015–2017.

Table 4 presents the number of projects and the values applied by Proadi-SUS according to the areas of activity established in Law No. 12.101/2009. For the characterization of the areas of activity, based on the data presented in Table 4, the reference number and value of the projects that had the exclusive indication in the area of activity were considered, added to the projects categorized in more than one area of activity, displaying the corresponding percentage.

**Table 4:** Proadi-SUS projects by area of activity, according to the number of projects carried out and amounts invested in the 3-year periods 2009–2011, 2012–2014 and 2015–2017.

Area of operation ^a^	2009–2011		2012–2014		2015–2017	
		
	Number of projects	Value in thousand *reais*	Number of projects	Value in thousand *reais*	Number of projects	Value in thousand *reais*
I	15	20,092	7	19,295	2	15,873
II	8	20,504	23	36,298	37	263,040
III	9	20,322	25	66,532	21	330,193
IV	52	332,988	34	194,209	21	62,440
V	14	86,373	19	205,077	17	340,779
I and II	1	94	4	16,917	4	35,479
I and III	1	1,165	8	4,063	0	0
I and IV	5	9,612	1	7,317	0	0
II and III	0	0	5	99,665	0	0
II and IV	15	69,120	16	81,137	11	222,411
III and IV	0	0	2	2,434	4	24,406
I, II and III	0	0	1	20,427	3	335,310
I, II and IV	0	0	0	0	1	31,676
I, III and IV	0	0	2	9,522	0	0
II, III and IV	0	0	0	0	1	6,881
All	4	39,984	2	252,035	1	5,786
No specification	0	0	0	0	11	87,971
	**124**	**600,258**	**149**	**1,014,934**	**134**	**1,762,251**

Source: Authors’ preparation based on data from the Ministry of Health.

Data in *reais*, deflated from the December 2017 IPCA.

^a^ Areas of activity established in Law No. 12.101/2009, where I refers to Technology Incorporation Evaluation studies; II = Human Resources Training; III = Research of Public Interest in Health; IV Development of Management Techniques and Operation in Health Services; and V Assistance Projects.

In the 3-year period 2009–2011, there was a significant amount of projects involving area of activity IV, with 61.3% being actions aimed at the Development of Management Techniques and Operation in Health Services, which corresponded to 75.3 % of resources for that period. When analyzing the period 2012–2014, projects involving area IV correspond to 38.3%, accounting for 53.9% of the resources utilized; and in the period 2015–2017, projects in the same area accounted for 29.1%, with a reference of 20.1% of resources. Thus, a reduction of actions in this area of activity and its respective funding is made explicit.

One area that saw growth in investment and actions was Area II, with projects aimed at Human Resources Training. In the 2009–2011 period, projects in this area accounted for 22.6%, with an amount of 21.6% of the tax exemption value; in the 2012–2014 period, they corresponded to 34.2% of the projects carried out, accounting for 49.9% of the tax exemption amount; and in the 2015–2017 period, they corresponded to 43.3% of the projects, accounting for 51.1% of the tax exemption value.

Both activity area I, Technology Incorporation Evaluation Studies, and activity area III, Research of Public Interest in Health, are related to research development. In the 2009–2011 period, projects linked to area I corresponded to 21%, accounting for 11.8% of the tax exemption amount; in the 2012–2014 period, they corresponded to 16.8% of the projects, accounting for 32.5% of the tax exemption amount; and in the 2015–2017 period, they corresponded to 8.2% of the projects, accounting for 24.1% of the tax exemption amount. It appears that the projects in this area of activity had significantly increased resources. The projects linked to area of activity III, in the 2009–2011 period, corresponded to 11.3% of the actions in an amount of 10.2% of the tax exemption value; in the 2012–2014 period, they represented 30.2% of the projects, accounting for 44.8% of the tax exemption amount; and in the 2015–2017 period, they corresponded to 22.4%, accounting for 39.9% of the tax exemption amount.

When analyzing the resources allocated to projects developed in area V, with assistance offers, a value of R$ 86.4 million (14.4% of resources) is verified for the period 2009–2011; R$205.1 million (20.2% of funds) for the 2012–2014 period; and R$340.8 million (19.3% of resources) for the period 2015–2017, totaling R$632.2 million (18.7% of total resources). When comparing the periods, there was a real increase of 57.9% in resources in this area of activity from the first to the second 3-year period and an increase of 39.8% in resources from the second to the third 3-year period. It is considered, however, that, because there was a divergence in the classification of the areas of activity, especially in the first three years in which this assistance area was not presented in a discriminating manner and many assistance projects were included in area of activity IV, it may be that some assistance project was already being developed previously but it was considered within some other area of activity.

## DISCUSSION

Data on the real growth of tax expenditures are corroborated by other studies in the area. Although there is a directing of resources to health policies through tax expenditures, it is important to highlight that the literature indicates that the understanding of SUS funding impasses must be articulated with the analysis of the institution of a restrictive macroeconomic policy in recent decades. There is a consideration that the tax relief measures adopted to combat the crisis affected, contradictorily, the financing of the social security and health budget, thus weakening the tax sources of social security, health and social assistance policies^16^.

Ocké-Reis^18^states that waiving reinforced the iniquity of the health system, which worsened the distribution of public spending *per capita* — direct and indirect — for lower and intermediate income strata. In addition, according to the author, pressure groups tended to preserve and exacerbate such iniquity and subsidies did not effectively relieve the medical and hospital services of the SUS, since users of health plans used public services in certain spheres, such as vaccination, urgency and emergency, blood bank, transplants, hemodialysis, high cost and technologically complex procedures. It should be noted, however, that funding for the SUS suffered considerable budgetary restrictions after 2016 with Constitutional Amendment No. 95/2016, which froze public spending for twenty years, being considered, contradictorily, as a possibility of mediation for the shortage of resources in the public health sector.

It can be considered that the continuous increase in tax expenditures is the result of the process of expanding the relationship between the state sphere and the business and philanthropic sectors in the 1990s and 2000s. It is evident that, in addition to assistance, philanthropic institutions considered strategic began to intervene in technology evaluation and incorporation studies, in human resources training and in research of public interest in health, in addition to the development of management techniques and operation in health services, spheres traditionally offered by the state sector.

Bahia^25^states that this regulation represented the clearest example at the time of the production of public policies to support the private sector, removing the conditionalities for granting a certificate of philanthropy for a subset of “cutting-edge” hospitals considered strategic. It was a measure aimed at helping hospitals that did not comply with the precept of serving at least 20% of SUS patients to maintain their tax benefits. This statement corroborates the analysis made in this article that this inflection is a milestone for the health sector and that it ends up favoring the business sector.

In view of the summaries of the projects, it can be observed that in the 2009-2011 period, actions focused mainly on highly complex procedures, but in the 2012-2015 period, in addition to these issues, some projects stood out regarding support for integrated regional health care systems and actions related to health surveillance protocols – projects that ended up expanding, in a certain way, the intervention, considering the scope of management techniques in the SUS. However, this explains that this large amount of committed resources does not necessarily dialogue with social demands and with the guidelines of public health policies.

In the few documents in which there is a qualitative analysis of the program, a weighting carried out in the Proadi-SUS 1st Cycle Evaluation Workshop^26^indicates that there is a continental dimension and regional heterogeneities in the country and that Proadi-SUS resources end up prioritizing certain regions. A contrast was also indicated in the Proadi-SUS projects with the demands of the Annual Management Report (RAG), whether referring to health indicators or demands, such as the language and conceptual terms used in both materials. The incorporation of management tools from the private sector is evident when they are implemented and incorporated into social policies, with the strengthening of entrepreneurship and capillarization of the actions of the business community in the social work field, with a conflict of interests between the private sector and the public sector.

The summaries of the projects indicate that training took place mainly in areas that demand high technology, especially in the first 3-year periods. The report by the Proadi-SUS 1st Cycle Assessment Workshop^26^also indicates that the definition of training priorities should be done at the regional level, through articulation between the Teaching-Service Integration Commission (Cies) and the Regional Intermanagement Commissions (CIR) of the SUS, with greater articulation between the definition of hospital offers and existing needs. This weighting is highlighted considering that the projects in this area were the ones that had the greatest growth in actions and resources between the three periods analyzed.

In addition, an observation made in the 2017 audit by the Federal Court of Auditors (TCU)^a^ on projects in the assistance area is that the MH should assess the costs of project procedures in order to have a cost reference, comparing the values proposed in the projects with the SUS table, in order to avoid the approval of actions whose procedures could be contractualized outside the program for lower amounts.

The TCU audit report cites a case for the 2015–2017 period of a percentage difference between the Proadi-SUS price and the SUS table of 536% in colonoscopy procedures, 392% in bariatric surgery, 203% in ultrasound examination, 152% in bone densitometry, and 101% in CT scans. It also mentions another situation, for the 2015-2017 period, in which there was a percentage difference between the Proadi-SUS price and the SUS table of 594% for the spirometry test^a^.

## FINAL CONSIDERATIONS

The Proadi-SUS has completed 12 years since its implementation and there is a lack of academic studies on it. It is also verified that this program represents the consolidation of a new form of philanthropy, since no previous experiences like this were found in the state management of the health sector. These considerations reinforce the need for more academic studies in the public health field. In addition, the literature indicates that it is urgent to analyze tax expenditures and how they impact SUS funding.

Proadi-SUS is also a program that establishes a different expression of the public-private partnership in the health sector enabling a new partnership configuration, such as hospitals considered strategic in line with the principles of the *new public management*. Proadi-SUS benefits few institutions and there is some inequity in the distribution of resources, with concentration in the Southeast region.

Based on this analysis, a real growth in general tax expenditures and in tax expenditures related to the philanthropic sector was verified in the last two decades. There was also a real increase in the program’s resources, when analyzing the 3-year periods 2009–2011, 2012–2014 and 2015–2017. There is a 40% variation from the first to the second period and a 42% variation from the second to the third period.

It is not possible to carry out a qualitative analysis of the use of these resources and the impacts of projects on the SUS due to insufficient data available. There are references in the materials published about the program^26^ and in the minutes of the Management Committee and the Evaluation Committee made available on the MH^1^website about the difficulty in monitoring and following up on projects, with little specificity about evaluation criteria, predefined indicators, and no submission of projects’ accountability reports by the hospitals that make up the program^a^. In addition to this question, general data on the results of the projects that appear published in some documents were not shared for the purposes of this research, although they were requested.

However, it is fearful to join a program where resources are concentrated in a few institutions. In addition, there are documents that refer to discrepancies in the projects carried out and in health indicators and demands presented in institutional documents. There is the incorporation of management tools from the private sector and a type of care linked to the logic of the market that do not necessarily match social and health demands.

As a development for future investigations, a qualitative characterization of the projects developed and the impact of the actions on the public sector demands is necessary.

## Footnotes

a Tribunal de Contas da União. Relatório de Auditoria TC 016.264/2017-7. Brasília DF; 2017.
