# Supplementary material for: Proadi-SUS: analysis of financial resources in the three-year periods 2009–2011, 2012–2014 and 2015–2017
Source: Rev Saude Publica. 2023 Sep 14;57:61. doi: 10.11606/s1518-8787.2023057004385 (PMC10519681; doi:10.11606/s1518-8787.2023057004385)
Supplement: Translation

# Translation: Proadi-SUS: análise dos recursos financeiros nos triênios 2009–2011, 2012–2014 e 2015–2017

**Authors:** Julia Amorim Santos, Luciana Palhares, Aquilas Mendes

**Affiliations:** I Universidade de São Paulo Faculdade de Saúde Pública Programa de Pós-Graduação em Saúde Pública São Paulo SP Brasil Universidade de São Paulo. Faculdade de Saúde Pública. Programa de Pós-Graduação em Saúde Pública. São Paulo, SP, Brasil; II Universidade Estadual de Campinas Faculdade de Educação Programa de Pós-Graduação em Sociologia da Educação Campinas SP Brasil Universidade Estadual de Campinas. Faculdade de Educação. Programa de Pós-Graduação em Sociologia da Educação. Campinas, SP, Brasil; III Pontifícia Universidade Católica de São Paulo Programa de Estudos Pós-graduados em Economia Política São Paulo SP Brasil Pontifícia Universidade Católica de São Paulo. Programa de Estudos Pós-graduados em Economia Política. São Paulo, SP, Brasil

**Keywords:** Política de Saúde, Parcerias Público-Privadas, Isenção Fiscal

## Abstract

**OBJETIVO:**

Caracterizar os recursos de isenção fiscal usufruídos no Programa de Apoio ao Desenvolvimento Institucional do Sistema Único de Saúde (Proadi-SUS) nos triênios 2009–2011, 2012–2014, 2015–2017, considerando o volume total dos recursos atrelado ao debate dos gastos tributários em saúde e a constituição de uma “nova modalidade de filantropia” no setor.

**MÉTODOS:**

Para compreender o setor filantrópico, analisaram-se os gastos tributários no período de 2001 e 2017. Para avaliar os recursos utilizados no programa, foram examinados os valores dos projetos e das áreas de atuação.

**RESULTADOS:**

Foi constatado um crescimento real dos valores dos gastos tributários gerais e dos gastos tributários referentes ao setor filantrópico. Constatou-se, também, um aumento real dos recursos do programa. Foram realizados 407 projetos, totalizando um valor de R$ 3,4 bilhões para o período. Ao se analisar o valor médio dos projetos, explicita-se ampliação dos valores para todos os hospitais inseridos no programa, com exceção de uma das instituições. Nos triênios 2009–2011 e 2012–2014, a área de atuação com maior número de projetos e mais recursos foi a “Área de técnicas e operação de gestão em serviços de saúde”. Já no triênio 2015–2017, o setor que contou com mais investimentos e maior número de projetos desenvolvidos foi o de “Capacitação de recursos humanos”.

**CONCLUSÃO:**

O programa caracteriza outro patamar na relação do setor público com o setor privado/empresariado da saúde, atrelado aos princípios da nova gestão pública. Faz-se necessária, como desdobramento para futuras investigações, uma caracterização qualitativa dos projetos desenvolvidos e do impacto das ações diante das demandas do setor público.

## INTRODUÇÃO

Segundo o Ministério da Saúde (MS), o Programa de Apoio ao Desenvolvimento Institucional do Sistema Único de Saúde (Proadi-SUS) é uma política dirigida ao fortalecimento do Sistema Único de Saúde (SUS) a ser conduzida em parceria com hospitais filantrópicos de qualidade reconhecida^1,2^. O financiamento desse programa se dá a partir dos gastos tributários, que são considerados gastos indiretos de isenção fiscal do Governo Federal. A política de gastos tributários visa atender a determinados objetivos econômicos e sociais e constitui uma exceção ao Sistema Tributário de Referência. O governo cede a desoneração de receitas tributárias para estimular determinadas áreas, consideradas essenciais, reduzindo a arrecadação potencial, mas, consequentemente, aumentando a disponibilidade econômica do contribuinte^3^.

O princípio do arranjo institucional para implementação do Proadi-SUS foi estabelecido nos anos 1990, durante o governo do presidente Fernando Henrique Cardoso. Por meio do Decreto nº 2.536, de 1998, estabeleceu-se pela primeira vez no setor da saúde a possibilidade de entidades filantrópicas acessarem isenções fiscais por meio da realização de projetos voltados ao SUS. Tais iniciativas vão além da oferta de serviços assistenciais como estabelecido pela filantropia tradicional regulamentada pela Lei nº 8.742, de 1993, que prevê oferta de 60% dos recursos em ações assistenciais para o SUS. Foi instituída, a partir desse momento, a possibilidade de desenvolvimento de projetos em outras áreas de atuação, limitada a 30% do valor usufruído com ações assistenciais^2^. Documento publicado pelas instituições que compõem o programa fazem referência ao surgimento de um novo patamar na relação entre os hospitais e a esfera estatal, com a instituição de uma nova modalidade de filantropia^4^.

Essa forma de filantropia, além de redimensionar a porcentagem de ações assistenciais como já referido, prediz também uma diferença nos parâmetros de custo. Na filantropia tradicional, tem-se como base de preço a tabela SUS, enquanto o Proadi-SUS permite que os hospitais executem as ações a partir da tabela de mercado.

Além das contribuições com recursos para atividades assistenciais e da ampliação de ações com a realização de projetos, pode-se afirmar que essa experiência se caracteriza como uma “nova modalidade de filantropia” por estar em consonância ao recomendado pela Reforma do Estado de 1995, que propunha mudanças na administração estatal e novas propostas gerenciais^5^, além da introdução de novos critérios para a concessão do certificado de filantropia para hospitais que foram considerados estratégicos. Institui-se um arranjo institucional com nova modalidade de relação público-privada com a regulamentação do empresariado para desenvolver trabalho social no setor da saúde^6,7^.

A literatura indica também que nos anos 2000/2010, inclusive por meio de orientações de organismos internacionais como o Banco Mundial, houve a ampliação da lógica gerencial e o relacionamento entre os diversos atores para financiar, monitorar, prestar e usar serviços de saúde. Atrelou-se o conceito de “empreendedorismo social” nas estratégias de políticas públicas e parte da responsabilidade pela implementação das políticas do Estado foi atribuída a outros setores que não o estatal, como o empresariado^8,9^. Portanto, observa-se consonância das diretrizes do programa com a racionalidade da *nova gestão pública*^10,11^. Um dos enfoques centrais estava voltado à adaptação e à transferência dos conhecimentos gerenciais desenvolvidos no setor privado para o estatal.

Embora a garantia de isenções fiscais por meio da realização de projetos tenha sido instituída no fim dos anos 1990, a regulamentação para o início das atividades de projetos de apoio ao SUS pelas entidades filantrópicas só foi consolidada em 2006, em diálogo com o avanço da lógica gerencial no setor após os anos 2000. O avanço dessa política para o setor da saúde foi marcado, também, pela nova regulamentação do Certificado de Entidades Beneficentes de Assistência Social (Cebas), em 2006, por meio do Decreto nº 5.895. Esse decreto regulamentou uma prática que vinha sendo realizada há oito anos, em que alguns hospitais considerados estratégicos já gozavam da isenção fiscal por meio desse outro por modelo de filantropia^2^. Assim, cabe destacar que uma das justificativas para a criação do Proadi-SUS se deu diante da necessidade de maior regulação pelo setor estatal em relação a essa nova forma de filantropia.

O programa foi instituído pela Lei nº 12.101/2009, que dispõe sobre o Cebas e regula os procedimentos de isenção de contribuições sociais. Foi a partir desse processo que se regulamentou a elaboração de projetos de apoio ao desenvolvimento institucional do SUS nas seguintes áreas de atuação: I — área de estudos de avaliação de incorporação de tecnologia; II — área de capacitação de recursos humanos; III — área de pesquisas de interesse público em saúde; e IV — área de técnicas e operação de gestão em serviços de saúde^1,2,12^.

Assim, o Proadi-SUS se configurou como uma experiência pioneira nesse formato, não sendo encontrados programas como esse na gestão estatal do setor da saúde anteriormente. No entanto, a partir de 2012, verificou-se a instituição de programas em formato semelhante, como o Programa Nacional de Apoio à Atenção Oncológica (Pronon) e o Programa Nacional de Apoio à Atenção da Saúde da Pessoa com Deficiência (Pronas/PCD)^13^, que também concede certificação de filantropia e isenção fiscal por meio da execução de projetos em outras áreas, além da assistência. Esse fato pode ser considerado uma justificativa em destaque para o desenvolvimento de investigações acadêmicas, além da constatação de que há uma escassez de análises do programa^14,15^.

Somada às justificativas diretamente relacionadas ao programa, há também a indicação na literatura da importância de análises sobre renúncia fiscal e gastos tributários em saúde, sendo novamente verificada certa escassez de publicações sobre essa temática^16^. Esse tópico é ainda mais urgente por estar atrelado à problemática do financiamento do SUS e ao combate do desfinanciamento no setor público, intensificado a partir de 2016, com a Emenda Constitucional nº 95^19,20^.

Salvador^17^ refere que na contemporaneidade o fundo público viabiliza a reprodução do capital e o estudo do orçamento deve ser considerado um elemento importante para compreender a política social. Para o autor, além de um aspecto técnico, o financiamento público reflete a correlação de forças sociais e os interesses envolvidos na apropriação dos recursos estatais. Dessa maneira, analisar o financiamento do Proadi-SUS deve perpassar também por uma investigação mais ampla das políticas sociais.

Este artigo tem como objetivo caracterizar os recursos de isenção fiscal usufruídos no Proadi-SUS nos triênios de 2009–2011, 2012–2014 e 2015–2017. A análise desses dados representa um recorte da pesquisa de doutorado que teve como objetivo examinar o programa em questão. Na tese de doutorado, apresenta-se um estudo que abarca articulação do programa junto às políticas sociais de maneira mais ampla^21^, no entanto, essa análise não está apresentada no escopo deste artigo.

## MÉTODOS

Foram solicitadas informações sobre o Proadi-SUS para o MS referentes aos projetos dos seus três primeiros triênios (2009–2011, 2012–2014 e 2015–2017). Tal solicitação foi dirigida tanto ao Departamento de Economia da Saúde, Investimentos e Desenvolvimento (Desid) — responsável pela gestão do programa — como por meio do Portal da Transparência da Controladoria-Geral da União (CGU). Disponibilizaram-se dados sobre os projetos em planilhas enviadas à pesquisadora e por meio de atualização do site do MS^1^. Como foram compartilhados materiais estruturados de diferentes maneiras, foi necessário um extenso trabalho de organização das informações para a realização da análise.

Para sistematização dos projetos, consideraram-se os nomes, sua descrição, as áreas de atuação e os valores aprovados ou executados, a depender de qual informação foi disponibilizada. Estabeleceu-se o seguinte levantamento para a análise, conforme os dados disponibilizados por meio das planilhas que apresentaram os recursos financeiros: *valores aprovados* para cada projeto apresentado nos triênios 2009–2011 e 2012–2014; *valores executados* relacionados aos projetos assistenciais do triênio 2012–2014; e *valores executados* relacionados aos projetos do triênio 2015–2017.

Após tratamento das informações, perceberam-se incongruências e divergências nos dados disponibilizados. Na planilha do triênio 2015–2017, não havia a especificação dos hospitais que realizaram cada projeto, sendo apresentados apenas o valor e o nome do projeto. Assim, foi necessário ainda cruzar as informações com a descrição dos projetos divulgados no site do Ministério da Saúde. Como alguns projetos também não estavam identificados nesse site, foi preciso buscar a referência do hospital por meio das atas do Comitê Gestor disponibilizadas no site institucional do MS^1^ ou na publicação Proadi-SUS^4^, realizada pelos hospitais que participam do programa. Os projetos divulgados no site do MS, mas que estavam indicados na planilha dos gastos financeiros como não executados, foram desconsiderados. Como havia uma imprecisão de nomenclatura nesses materiais, consideraram-se como mesmo projeto aqueles com títulos e objetivos semelhantes.

Os pesquisadores encontraram divergências também nas referências das áreas de atuação, quando comparadas às informações do site do MS e da planilha de valores dos projetos. Como indicado, utilizou-se como base para a análise a planilha dos recursos financeiros, mas para identificar as divergências referentes à área de atuação foi realizada a leitura da descrição do projeto, fazendo ajustes a partir dessa análise.

Foram sistematizados também os gastos e as despesas tributárias vinculados ao setor filantrópico no período de 2001 a 2017. Essas informações foram acessadas a partir do Demonstrativo dos Gastos Tributários disponibilizado pelo Ministério da Fazenda no site da Receita Federal^22^. Todos os dados referentes aos valores correntes foram atualizados em valores reais, utilizando o deflator do Índice Nacional de Preços ao Consumidor Amplo (IPCA) de dezembro de 2017. Considerou-se esse período de referência por ser o último ano incluso no período de análise deste trabalho.

## RESULTADOS

Para uma análise mais detalhada sobre o crescimento do setor filantrópico nos últimos anos, apresentam-se os dados dos gastos tributários para o período de 2001 e 2017 (Tabela 1).

**Tabela 1:** Gastos tributários gerais do Governo Federal e gastos vinculados ao setor filantrópico no período entre 2001 e 2017, considerando a variação percentual anual.

Ano	Total de gasto tributário		Total de gasto vinculado ao setor filantrópico	
	
	Valor em milhares de reais	Variação percentual anual (%)	Valor em milhares de reais	Variação percentual anual (%)
2001	845.723		8.854	
2002	9.650.942	91,2	15.488	42,8
2003	10.864.218	11,2	96.335	83,9
2004	11.813.505	8	896.402	89,3
2005	16.135.205	26,8	1.693.394	47,1
2006	22.605.381	28,6	2.329.187	27,3
2007	29.302.590	22,9	3.409.711	31,7
2008	44.751.559	34,5	3.857.260	11,6
2009	62.578.136	28,5	4.789.882	19,5
2010	74.023.452	15,5	6.346.147	24,5
2011	80.365.722	7,9	5.887.764	-7,8
2012	106.962.736	24,9	6.928.435	15
2013	131.939.897	18,9	8.330.175	16,8
2014	206.245.036	36	17.885.947	53,4
2015	258.120.379	20,1	21.826.574	18,1
2016	263.247.117	1,9	23.478.916	7
2017	284.846.251	7,6	26.194.656	10,4

Fonte: Elaboração própria com base em dados da Receita Federal (2017).

Dados em reais, deflacionados a partir do IPCA de dezembro de 2017.

Observa-se constante crescimento dos valores de ano a ano, com aumento mais elevado dos gastos tributários gerais do Governo Federal de 2003 a 2007. Ao analisar os gastos tributários referentes ao setor filantrópico, também houve crescimento significativo e constante, com exceção da passagem de 2010 para 2011, que contou com uma queda de 7,8% do valor, mas, em compensação, teve aumento de 15% no ano seguinte. Ainda referente à Tabela 1, examinando os gastos tributários relacionados ao setor da filantropia, constata-se um aumento significativo no início dos anos 2000. Os valores referentes aos gastos tributários relacionados à filantropia também tiveram um aumento expressivo entre 2012 e 2015, com destaque para o ano de 2014, que contou com uma variação percentual de 53,4%. Ao se considerar as funções orçamentárias em relação aos gastos tributários, também houve a constatação de um aumento real dos recursos para a função da assistência social, da ciência e tecnologia, da cultura, do esporte e lazer, da educação e da saúde.

Os seis hospitais que compuseram o Proadi-SUS no período analisado são: Hospital Alemão Oswaldo Cruz (HAOC – SP); Hospital do Coração (HCor – SP); Hospital Israelita Albert Einstein (HIAE – SP); Hospital Moinhos de Vento (HMV – RS); Hospital Samaritano (SP); Hospital Sírio Libanês (HSL – SP). A partir da análise dos dados dos projetos desenvolvidos e dos valores de isenção fiscal vinculados ao Proadi-SUS, constatou-se que, nos três triênios, foram realizados 407 projetos, totalizando um valor de R$ 3,4 bilhões (Tabela 2).

**Tabela 2:** Indicação do número de projetos e dos valores de isenção fiscal de cada uma das instituições que compõem o Proadi-SUS, considerando os triênios 2009–2011, 2012–2014 e 2015–2017, e referência total para o período estudado.

Nome da Instituição	Triênio 2009–2011		Triênio 2012–2014		Triênio 2015–2017	
		
	Número de projetos	Valor em milhares de reais	Número de projetos	Valor em milhares de reais	Número de projetos	Valor em R$
Hospital Alemão Oswaldo Cruz	15	66.556	17	107.834	18	164.029
Hospital do Coração	27	50.103	33	88.671	28	126.683
Hospital Israelita Albert Einstein	32	274.780	41	442.417	23	727.123
Hospital Moinhos de Vento	4	50.530	7	105.928	18	188.689
Hospital Samaritano	21	33.473	26	88.711	14	123.569
Hospital Sírio Libanês	25	124.814	25	181.371	33	432.156
**Total por triênio**	**124**	**600.258**	**149**	**1.014.934**	**134**	**1.762.251**
**Total do programa**	**407 projetos**					
	**3.377.444**					

Fonte: Elaboração própria com base em dados do Ministério da Saúde.

Dados em reais, deflacionados a partir do IPCA de dezembro de 2017.

Como pode ser observado na Tabela 2, no primeiro triênio, no período de 2009 a 2011, foram desenvolvidos 124 projetos, considerando recursos de isenção fiscal no valor de R$ 600,3 milhões. Para o segundo triênio, no período de 2012 a 2014, foram criados 149 projetos, considerando recursos de isenção fiscal no valor de R$ 1 bilhão. Para o terceiro triênio, no período de 2015 a 2017, foram elaborados 134 projetos, considerando recursos de isenção fiscal no valor de R$ 1,8 bilhão. Constata-se um aumento real dos recursos do programa, havendo uma variação percentual anual de 40,9% do primeiro triênio para o segundo e uma variação percentual anual de 42,4% do segundo triênio para o terceiro.

Ao analisar o número de projetos e recursos usufruídos por cada um dos hospitais em cada triênio, identifica-se uma diferença significativa de recursos utilizados e quantidade de projetos desenvolvidos por cada uma das instituições.

Considerando o montante total usufruído por cada um dos hospitais, observa-se que 42,8% dos recursos corresponderam aos projetos do Hospital Israelita Albert Einstein e 21,9% dos recursos eram referentes aos projetos do Hospital Sírio Libanês. Reforça-se que o valor de isenção fiscal é calculado a partir da receita de cada hospital ao ano. Essa proporção não é correspondente quando comparada à análise do número de projetos realizados. Adotando como base os dois hospitais referidos, nota-se que 23,6% corresponderam aos projetos do Hospital Israelita Albert Einstein e 20,4% aos projetos do Hospital Sírio Libanês. Explicita-se, assim, uma diferenciação entre os custos médios dos projetos.

Ao discriminar os valores de isenção fiscal por triênio do programa, observa-se que todos os hospitais tiveram uma ampliação real nos recursos, com destaque para a expansão dos hospitais Israelita Albert Einstein e Sírio Libanês. Analisando o valor médio dos projetos de cada hospital, observa-se um aumento dos valores dos projetos para todos os hospitais ao comparar o primeiro, o segundo e o terceiro triênio, com exceção do Hospital Moinhos de Vento, que teve uma queda no valor médio do segundo para o terceiro triênio (Tabela 3).

**Tabela 3:** Valor médio de projeto de cada uma das instituições que compõem o Proadi-SUS, considerando os triênios 2009–2011, 2012–2014 e 2015–2017, e referência total para o período estudado.

Nome da instituição	Triênio 2009–2011	Triênio 2012–2014	Triênio 2015–2017	Total
			
	Valor em milhares de reais	Valor em milhares de reais	Valor em milhares de reais	Valor em milhares de reais
Hospital Alemão Oswaldo Cruz	4.437	6.343	9.112	6.768
Hospital do Coração	1.855	2.687	4.524	3.016
Hospital Israelita Albert Einstein	8.586	10.790	31.614	15.045
Hospital Moinhos de Vento	12.632	15.132	10.482	11.901
Hospital Samaritano	1.593	3.411	8.826	4.028
Hospital Sírio Libanês	4.992	7.254	13.095	8.895

Fonte: Elaboração própria com base em dados do Ministério da Saúde.

Dados em reais, deflacionados a partir do IPCA de dezembro de 2017.

Fica explícito que o valor médio dos projetos do Hospital Israelita Albert Einstein é maior do que o dos demais hospitais. Com relação aos recursos do primeiro triênio, a isenção fiscal desse hospital correspondeu a 45,5% do recurso disponível no período, realizando 25,8% dos projetos executados. Quanto aos recursos do segundo triênio, a isenção fiscal desse hospital correspondeu a 43,6% do recurso disponível no período, realizando 27,5% dos projetos executados. Referente aos recursos do terceiro triênio, a isenção fiscal desse hospital correspondeu a 41,3% do recurso disponível no período, realizando 17,2% dos projetos executados. Portanto, os valores médios dos projetos no terceiro triênio foram significativamente maiores em comparação com os outros períodos, com uma variação percentual de 72,8% do valor médio dos projetos do primeiro para o terceiro triênio.

Cabe destacar também uma diferença significativa da porcentagem dos recursos e do número de projetos realizados pelo Hospital Moinhos de Vento, com uma ampliação significativa do número de projetos desse hospital ao comparar o terceiro triênio com o primeiro.

É observada proximidade entre a porcentagem de projetos e a porcentagem de recursos do Hospital Alemão Oswaldo Cruz e do Hospital Sírio Libanês. Os hospitais que tiveram o menor valor médio de projeto foram o Hospital do Coração e o Hospital Samaritano.

Foi aberta a possibilidade, ainda, a partir do segundo triênio, de que os hospitais integrantes do programa, e porventura candidatos ao financiamento pelo Banco Nacional de Desenvolvimento Econômico e Social (BNDES), pudessem aplicar 5% dos seus financiamentos por meio do desenvolvimento de projetos vinculados ao Proadi-SUS. Portanto, além dos projetos do Proadi-SUS apresentados, foi estabelecida uma vinculação de novos projetos com o BNDES. Na planilha de recursos do triênio 2012–2014 referente aos projetos atrelados ao BNDES, foi identificada a execução de 11 projetos, num montante correspondente a R$ 19,4 milhões (valores executados). Dos hospitais que compõem o programa, apenas o Hospital Israelita Albert Einstein aderiu a essa modalidade nesse triênio. Foi possível identificar, por meio das atas do Comitê Gestor, a existência de seis projetos desenvolvidos pelo Hospital Israelita Albert Einstein e pelo Hospital Sírio Libanês nessa modalidade, com valores aprovados num montante de R$ 28 milhões para o triênio de 2015–2017.

Na Tabela 4 apresentam-se o número de projetos e os valores aplicados do Proadi-SUS conforme áreas de atuação estabelecidas na Lei nº 12.101/2009. Para a caracterização das áreas de atuação, a partir dos dados apresentados na Tabela 4, foram considerados a referência do número e o valor dos projetos que tinham a indicação exclusiva na área de atuação, somados aos projetos categorizados em mais de uma área de atuação, apresentando a porcentagem correspondente.

**Tabela 4:** Projetos Proadi-SUS conforme área de atuação, segundo a quantidade de projetos realizados e valores aplicados nos triênios 2009–2011, 2012–2014 e 2015–2017.

Área de atuação^a^	Triênio 2009–2011		Triênio 2012–2014		Triênio 2015–2017	
		
	Número de projetos	Valor em milhares de reais	Número de projetos	Valor em milhares de reais	Número de projetos	Valor em milhares de reais
I	15	20.092	7	19.295	2	15.873
II	8	20.504	23	36.298	37	263.040
III	9	20.322	25	66.532	21	330.193
IV	52	332.988	34	194.209	21	62.440
V	14	86.373	19	205.077	17	340.779
I e II	1	94	4	16.917	4	35.479
I e III	1	1.165	8	4.063	0	0
I e IV	5	9.612	1	7.317	0	0
II e III	0	0	5	99.665	0	0
II e IV	15	69.120	16	81.137	11	222.411
III e IV	0	0	2	2.434	4	24.406
I, II e III	0	0	1	20.427	3	335.310
I, II e IV	0	0	0	0	1	31.676
I, III e IV	0	0	2	9.522	0	0
II, III e IV	0	0	0	0	1	6.881
Todas	4	39.984	2	252.035	1	5.786
Sem especificação	0	0	0	0	11	87.971
	**124**	**600.258**	**149**	**1.014.934**	**134**	**1.762.251**

Fonte: Elaboração própria com base em dados do Ministério da Saúde.

Dados em reais, deflacionados a partir do IPCA de dezembro de 2017.

^a^ Áreas de atuação estabelecidas na Lei nº 12.101/2009, sendo I referente a Estudos de Avaliação e Incorporação de Tecnologias; II referente a Capacitação de Recursos Humanos; III referente a Pesquisas de Interesse Público em Saúde; IV referente a Desenvolvimento de Técnicas e Operação de Gestão em Serviços de Saúde; e V referente a Projetos Assistenciais.

No triênio 2009–2011, verifica-se um montante significativo de projetos envolvendo a área de atuação IV, sendo que 61,3% foram ações voltadas para Desenvolvimento de Técnicas e Operação de Gestão em Serviços de Saúde, o que correspondeu a 75,3% dos recursos daquele triênio. Quando se analisa o triênio 2012–2014, os projetos envolvendo a área de atuação IV correspondem a 38,3% referentes a 53,9% dos recursos usufruídos; e no triênio 2015–2017 os projetos nessa mesma área correspondem a 29,1% com referência de 20,1% dos recursos. Explicita-se, assim, uma redução das ações nessa área de atuação e no seu respectivo financiamento.

Uma área que contou com crescimento de investimento e ações foi a área de atuação II, com projetos voltados à Capacitação de Recursos Humanos. No triênio 2009–2011, os projetos dessa área corresponderam a 22,6%, com montante de 21,6% do valor de isenção fiscal; no triênio 2012–2014, corresponderam a 34,2% dos projetos realizados referentes a 49,9% do valor de isenção fiscal; e no triênio 2015–2017 corresponderam a 43,3% dos projetos, referentes a 51,1% do valor da isenção fiscal.

Tanto a área de atuação I, Estudos de Avaliação e Incorporação de Tecnologias, como a área de atuação III, Pesquisas de Interesse Público em Saúde, estão relacionadas com o desenvolvimento de pesquisa. No triênio 2009–2011, os projetos vinculados à área de atuação I corresponderam a 21%, referentes a 11,8% do valor de isenção fiscal; no triênio 2012–2014, corresponderam a 16,8% dos projetos, relativos a 32,5% do valor de isenção fiscal; e no triênio 2015–2017 corresponderam a 8,2% dos projetos, referentes a 24,1% do valor de isenção fiscal. Verifica-se que os projetos nessa área de atuação tiveram recursos significativamente aumentados. Já os projetos vinculados à área de atuação III, no triênio 2009–2011, corresponderam a 11,3% das ações num montante de 10,2% do valor de isenção fiscal; no triênio 2012–2014, representaram 30,2% dos projetos num montante de 44,8% do valor de isenção fiscal; e no triênio 2015–2017, corresponderam a 22,4%, referentes a 39,9% do valor de isenção fiscal.

Quando se analisa os recursos destinados aos projetos desenvolvidos na área de atuação V, com ofertas assistenciais, constata-se um valor de R$ 86,4 milhões (14,4% dos recursos) para o triênio 2009–2011; R$ 205,1 milhões (20,2% dos recursos) para o triênio 2012–2014; e R$ 340,8 milhões (19,3% dos recursos) para o triênio 2015–2017, totalizando um valor de R$ 632,2 milhões (18,7% dos recursos totais). Quando comparados os períodos, houve um aumento real de 57,9% dos recursos nessa área de atuação do primeiro para o segundo triênio e um aumento de 39,8% dos recursos do segundo triênio para o terceiro. Pondera-se, entretanto, que, por haver uma divergência de classificação das áreas de atuação, principalmente no primeiro triênio em que essa área assistencial não era apresentada de maneira discriminada e muitos projetos assistenciais estavam inclusos na área de atuação IV, pode ser que algum projeto assistencial já estivesse sendo desenvolvido anteriormente, mas foi considerado dentro de alguma outra área de atuação.

## DISCUSSÃO

Os dados de crescimento real dos gastos tributários são corroborados por outros estudos da área. Apesar de haver um direcionamento de recursos para as políticas de saúde por meio dos gastos tributários, é importante destacar que a literatura indica que a compreensão dos impasses do financiamento do SUS deve ser articulada com análise da instituição de uma política macroeconômica restritiva das últimas décadas. Há a ponderação de que as medidas de desoneração tributária adotadas para combater a crise afetaram, contraditoriamente, o financiamento do orçamento da seguridade social e da saúde, enfraquecendo, com isso, as fontes tributárias das políticas de previdência social, saúde e assistência social^16^.

Ocké-Reis^18^ afirma que a renúncia reforçou a iniquidade do sistema de saúde, o que piorou a distribuição do gasto público *per capita* — direto e indireto — para os extratos inferiores e intermediários de renda. Além disso, segundo o autor, os grupos de pressão tenderam a conservar e a agravar tal iniquidade e os subsídios não desafogaram efetivamente os serviços médico-hospitalares do SUS, visto que os usuários de planos de saúde utilizaram os serviços públicos em certas esferas, como vacinação, urgência e emergência, banco de sangue, transplantes, hemodiálise, procedimentos de alto custo e de complexidade tecnológica. Cabe ressaltar, no entanto, que o financiamento para o SUS sofreu considerável restrição orçamentária após 2016 com a Emenda Constitucional nº 95/2016, que congelou os gastos públicos por vinte anos, sendo considerada, contraditoriamente, como uma possibilidade de mediação para a escassez de recursos no setor público da saúde.

Pode-se considerar que o aumento contínuo dos gastos tributários é resultado do processo de ampliação da relação entre a esfera estatal e os setores empresarial e filantrópico nos anos 1990 e 2000. Fica evidente que, além da assistência, as instituições filantrópicas consideradas estratégicas passaram a intervir em estudos de avaliação e de incorporação de tecnologias, na capacitação de recursos humanos e em pesquisas de interesse público em saúde, além do desenvolvimento de técnicas e de operação de gestão em serviços de saúde, esferas tradicionalmente ofertadas pelo setor estatal.

Bahia^25^ afirma que essa regulamentação representou o exemplo mais claro à época da produção de políticas públicas de suporte ao setor privado, retirando as condicionalidades à concessão do certificado de filantropia de um subconjunto de hospitais de “ponta” considerados estratégicos. Foi uma medida voltada a auxiliar os hospitais que não cumpriam o preceito do atendimento de pelo menos 20% de pacientes do SUS a manter seus benefícios fiscais. Essa afirmação corrobora a análise feita neste artigo de que essa inflexão é um marco para o setor da saúde e que acaba privilegiando o setor empresarial.

Diante dos resumos dos projetos, pode-se observar que no triênio 2009–2011 as ações se voltaram principalmente para procedimentos de alta complexidade, mas no triênio 2012–2015, além dessas questões, destacaram-se alguns projetos referentes ao apoio a sistemas regionais de atenção integrada à saúde e ações referentes a protocolos da vigilância sanitária – projetos que acabaram ampliando, de certa maneira, a intervenção, considerando o âmbito de técnicas de gestão no SUS. No entanto, isso explicita que esse grande montante de recursos empenhados não dialoga, necessariamente, com as demandas sociais e com as diretrizes das políticas públicas de saúde.

Nos poucos documentos em que há uma análise qualitativa do programa, uma ponderação realizada na Oficina de Avaliação do 1º Ciclo Proadi-SUS^26^ indica que existe uma dimensão continental e heterogeneidades regionais no país e que os recursos do Proadi-SUS acabam priorizando determinadas regiões. Indicou-se, também, um contraste nos projetos do Proadi-SUS com as demandas do Relatório Anual de Gestão (RAG), seja referente aos indicadores ou às demandas de saúde, como o linguajar e os termos conceituais utilizados em ambos os materiais. Fica evidente a incorporação de ferramentas gerenciais do setor privado ao serem implementadas e incorporadas às políticas sociais, com fortalecimento do empreendedorismo e capilarização das ações do empresariado no campo do trabalho social, havendo um conflito de interesses entre o setor privado e o setor público.

Os resumos dos projetos indicam que a formação se deu, principalmente, considerando áreas que demandam alta tecnologia, sobretudo nos primeiros triênios. O relatório da Oficina de Avaliação do 1º Ciclo Proadi-SUS^26^ indica também que a definição de prioridades de formação deveria ser feita no âmbito regional, mediante articulação da Comissão de Integração Ensino-Serviço (Cies) com as Comissões Intergestoras Regionais (CIR) do SUS, com maior articulação entre a definição das ofertas dos hospitais e as necessidades existentes. Destaca-se essa ponderação considerando que os projetos nessa área foram os que contaram com maior crescimento de ações e recursos entre os triênios analisados.

Além disso, uma observação feita na auditoria do Tribunal de Contas da União (TCU) de 2017^a^ sobre os projetos da área de assistência é que o MS deveria avaliar os custos dos procedimentos dos projetos para ter um referencial de custo, comparando os valores propostos nos projetos com a tabela SUS, de modo a evitar a aprovação de ações cujos procedimentos poderiam ser contratualizados fora do programa por valores menores.

O relatório da auditoria do TCU cita um caso para o triênio 2015–2017 de uma diferença percentual entre o preço Proadi-SUS e a tabela SUS de 536% em procedimentos de colonoscopia, 392% em cirurgia bariátrica, 203% em exame de ultrassonografia, 152% em exame de densitometria óssea e 101% em exames de tomografia. Cita também outra situação, do triênio 2015–2017, em que se verificou uma diferença percentual entre o preço Proadi-SUS e a tabela SUS de 594% para exame de espirometria^a^.

## CONSIDERAÇÕES FINAIS

O Proadi-SUS completa 12 anos desde sua implementação e há a constatação de que faltam estudos acadêmicos sobre ele. Constata-se, também, que esse programa representa a consolidação de uma nova modalidade de filantropia, uma vez que não foram encontradas experiências prévias como essa na gestão estatal do setor da saúde. Essas considerações reforçam a necessidade de mais estudos acadêmicos na área da saúde pública. Além disso, a literatura indica que é urgente analisar os gastos tributários e como eles impactam o financiamento do SUS.

O Proadi-SUS é também um programa que institui outro patamar na relação entre os setores da saúde público, privado e empresariado, possibilitando uma nova configuração de parceria, como hospitais considerados estratégicos em consonância com os princípios da *nova gestão pública*. O Proadi-SUS beneficia poucas instituições e há uma inequidade na distribuição de recursos, com concentração na região Sudeste.

Foi constatado, a partir desta análise, um crescimento real dos gastos tributários gerais e dos gastos tributários referentes ao setor filantrópico nas duas últimas décadas. Constatou-se também um aumento real referente aos recursos do programa, quando analisados os triênios 2009–2011, 2012–2014 e 2015–2017. Verificam-se uma variação percentual de 40% do primeiro triênio para o segundo e uma variação percentual de 42% do segundo triênio para o terceiro.

Não é possível fazer uma análise qualitativa da utilização desses recursos e dos impactos dos projetos no SUS por insuficiência de dados disponibilizados. Há referências nos materiais publicados sobre o programa^26^ e nas atas do Comitê Gestor e do Comitê de Avaliação disponibilizadas no site do MS^1^ sobre dificuldade no monitoramento e acompanhamento dos projetos, com pouca especificidade sobre critérios de avaliação, indicadores predefinidos e não abertura da prestação de contas dos projetos pelos hospitais que compõem o programa^a^. Além dessa questão, não foram compartilhados para fins desta pesquisa, embora tenham sido solicitados, dados gerais de resultados dos projetos que aparecem publicados em alguns documentos.

É questionável aderir a um programa em que os recursos estão concentrados em poucas instituições. Além disso, existem documentos que referem discrepância nos projetos realizados e em indicadores e demandas de saúde apresentados em documentos institucionais. Há incorporação de ferramentas gerenciais do setor privado e uma modalidade de cuidado atrelada à lógica do mercado que não condizem necessariamente com as demandas sociais e de saúde.

Faz-se necessária, como desdobramento para futuras investigações, uma caracterização qualitativa dos projetos desenvolvidos e o impacto das ações diante das demandas do setor público.

## Footnotes

a Tribunal de Contas da União . Relatório de Auditoria TC 016.264/2017-7. Brasília DF; 2017.
